# Psychedelics reopen the social reward learning critical period

**DOI:** 10.1038/s41586-023-06204-3

**Published:** 2023-06-14

**Authors:** Romain Nardou, Edward Sawyer, Young Jun Song, Makenzie Wilkinson, Yasmin Padovan-Hernandez, Júnia Lara de Deus, Noelle Wright, Carine Lama, Sehr Faltin, Loyal A. Goff, Genevieve L. Stein-O’Brien, Gül Dölen

**Affiliations:** 1 The Solomon H. Snyder Department of Neuroscience, Johns Hopkins University, School of Medicine, Baltimore, MD USA; 2 The Brain Science Institute, Johns Hopkins University, School of Medicine, Baltimore, MD USA; 3 Department of Oncology, Division of Biostatistics and Bioinformatics, Sidney Kimmel Comprehensive Cancer Center, Baltimore, MD USA; 4 McKusick–Nathans Department of Genetic Medicine, Johns Hopkins University, School of Medicine, Baltimore, MD USA; 5 The Kavli Neuroscience Discovery Institute, Johns Hopkins University, School of Medicine, Baltimore, MD USA; 6 The Department of Neurology, Johns Hopkins University, School of Medicine, Baltimore, MD USA; 7 The Center for Psychedelics and Consciousness Research, Johns Hopkins University, School of Medicine, Baltimore, MD USA; 8 The Wendy Klag Institute for Autism and Developmental Disabilities, Johns Hopkins University, School of Medicine, Baltimore, MD USA

**Keywords:** Cooperation, Long-term memory, Social behaviour

## Abstract

Psychedelics are a broad class of drugs defined by their ability to induce an altered state of consciousness^1,2^. These drugs have been used for millennia in both spiritual and medicinal contexts, and a number of recent clinical successes have spurred a renewed interest in developing psychedelic therapies^3–9^. Nevertheless, a unifying mechanism that can account for these shared phenomenological and therapeutic properties remains unknown. Here we demonstrate in mice that the ability to reopen the social reward learning critical period is a shared property across psychedelic drugs. Notably, the time course of critical period reopening is proportional to the duration of acute subjective effects reported in humans. Furthermore, the ability to reinstate social reward learning in adulthood is paralleled by metaplastic restoration of oxytocin-mediated long-term depression in the nucleus accumbens. Finally, identification of differentially expressed genes in the ‘open state’ versus the ‘closed state’ provides evidence that reorganization of the extracellular matrix is a common downstream mechanism underlying psychedelic drug-mediated critical period reopening. Together these results have important implications for the implementation of psychedelics in clinical practice, as well as the design of novel compounds for the treatment of neuropsychiatric disease.

## Abstract (web summary)

Behavioural electrophysiological and transcriptomic studies in mice show that psychedelic drugs reopen the social reward learning critical period and suggest that this involves reorganization of the extracellular matrix.

## Main

Classically, psychedelics have been defined to include drugs such as lysergic acid diethylamide (LSD), mescaline, phenylcyclohexyl piperidine (PCP), ibogaine, 3,4-methylenedioxymethamphetamine (MDMA), psylocibin and ketamine, because each of these compounds produces alterations to sensory, self, time and space perception that are “so alien to everyday experience that they shed new light on the workings of these everyday mental functions”^1^. Although more recent attempts have been made to subcategorize psychedelics^10^ on the basis of the subjective character of the altered state that they induce (for example, hallucinogenic, empathogenic, oneirogenic or dissociative), their chemical structure (for example, tryptamines, phenethylamines or arylcyclohexamines), or their principal binding target (for example, serotonin receptor 2A (5-HT_2A_R), monoamine transporter, κ-opioid receptor (KOR) or *N*-methyl-d-aspartate receptor (NMDAR)), the importance of these categories for therapeutic applications remains unclear, since psychedelics that span the diversity of classification systems have shown remarkable promise for the treatment of addiction^4,5^, post-traumatic stress disorder^6,7^ (PTSD) and depression^3,8,9^. Thus, identification of a common neurobiological mechanism that can account for the shared therapeutic effects of psychedelics is an obvious priority for translational neuroscience.

During specific periods of brain development, the nervous system exhibits heightened sensitivity to ethologically relevant stimuli, as well as increased malleability for synaptic, circuit and behavioural modifications. These mechanistically constrained windows of time are called critical periods and neuroscientists have long sought methods to reopen them for therapeutic benefit. Recently, we have discovered a novel critical period for social reward learning and shown that the empathogenic psychedelic MDMA is able to reopen this critical period^11^. This mechanism shares a number of features with the therapeutic effects of MDMA-assisted psychotherapy for the treatment of PTSD, including rapid onset, durability and context dependence^6,7^. At the same time, cocaine does not reopen the social reward learning critical period^11^, and since cocaine does not share the psychedelics’ therapeutic profile^12^, these results lend further support for the view that the reinstatement of social reward learning in adulthood underlies the therapeutic efficacy of MDMA.

Whether the ability of MDMA to reopen the critical period for social reward learning generalizes across psychedelics remains an open question. MDMA is classified as an ‘empathogen’ because its acute subjective effects are distinctly prosocial in quality^13^. The fact that this quality is not shared by hallucinogenic psychedelics such as psilocybin and LSD^14^, dissociative psychedelics such as ketamine^15^, or oneirogenic psychedelics such as ibogaine^16^ challenges the idea that these drugs could reopen the social reward learning critical period. However, the psychotropic effects of MDMA include an altered state of consciousness shared by all psychedelics^1,2^, and if it is this characteristic rather than its prosocial properties that embodies the subjective experience of reopening critical periods, then the ability to reinstate social reward learning in adulthood might generalize across psychedelics.

## Critical period reopening is a shared property

**Fig. 1:**
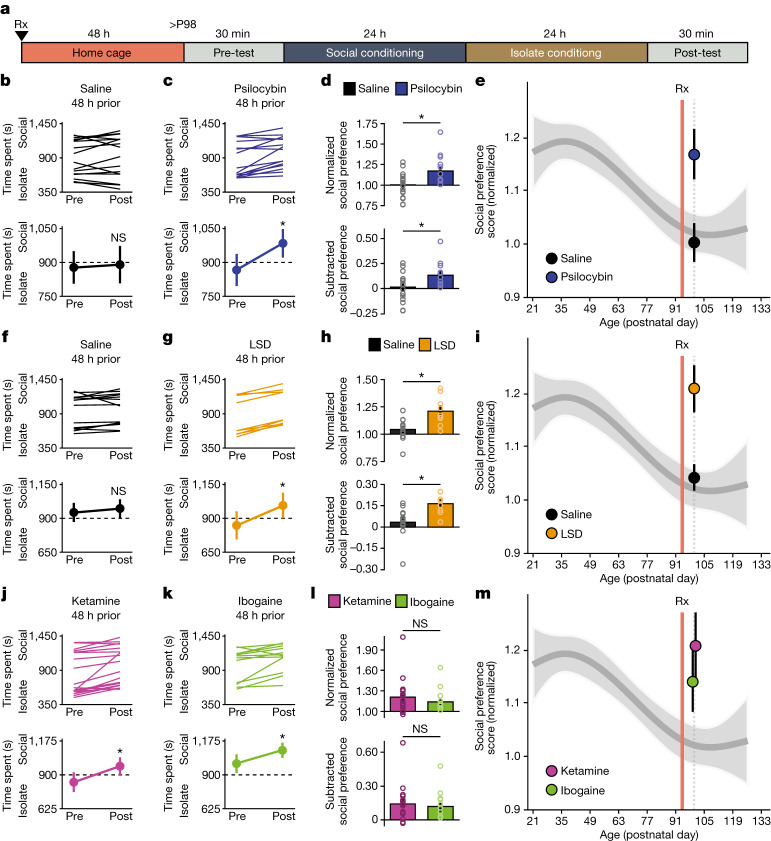
Psychedelics reopen the social reward learning critical period. **a**, Experimental time course of i.p. pretreatment (Rx) in sCPP. **b**,**c**,**f**,**g**,**j**,**k**, Individual (top) and average (bottom) responses of P98 mice indicate that mice pretreated with psilocybin (0.3 mg kg^−1^) (**c**; *n* = 15, *t*_(14)_ = −3.741, *P* = 0.002), LSD (1 µg kg^−1^) (**g**; *n* = 9, *t*_(8)_ = −7.095, *P* < 0.001), ketamine (3 mg kg^−1^) (**j**; *n* = 18, *t*_(17)_ = −3.826, *P* < 0.002), and ibogaine (40 mg kg^−1^) (**k**; *n* = 12, *t*_(11)_ = −2.690, *P* = 0.02) but not saline (**b**; *n* = 17 mice, *t*_(16)_ = −0.441, *P* = 0.665. **f**; *n* = 14 mice, *t*_(13)_ = −1.215, *P* = 0.25) develop a preference for the social bedding cue. Two-tailed paired *t*-test. **d**,**h**,**l**, Comparisons reveal a significant increase in normalized (top) and subtracted (bottom) social preference for pretreatment with psilocybin versus saline (**d**; normalized, *t*_(30)_ = −2.800, *P* = 0.009; subtracted, *t*_(30)_ = −2.401, *P* = 0.023), and with LSD versus saline (**h**; normalized, *t*_(21)_ = −3.558, *P* = 0.002; subtracted, *t*_(21)_ = −3.344, *P* = 0.003), but no difference between pretreatment with ketamine and ibogaine (**l**; normalized, *t*_(28)_ = 0.749, *P* = 0.460; subtracted, *t*_(28)_ = 0.409, *P* = 0.686). Two-tailed unpaired *t*-test, with Welch’s correction to account for unequal variance in **l** subtracted. *P < 0.05; NS, not significant (*P* > 0.05). **e**,**i**,**m**, Normalized social preference in mice pretreated with psilocybin versus saline (**e**), LSD versus saline (**i**) and ibogaine versus ketamine (**m**), plotted against a natural spline regression model of the developmental time course of normalized social preference scores. Comparison with the natural spline model revealed that the magnitude of sCPP in saline-treated mice did not deviate significantly from the closed state (**b**; *P* = 0.72) (**f**; *P* = 0.90), whereas mice pretreated with psilocybin (*P* = 1.12 × 10^−6^), LSD (*P* = 1.76 × 10^−9^), ketamine (*P* = 8.78 × 10^−4^) or ibogaine (*P* = 3.17 × 10^−5^) demonstrated a significant mean shift in range of the open state. Comparisons with the natural spline model were considered not significant (*P* > 0.1). Rx indicates drug treatment. Data are as mean ± s.e.m. *n* refers to the number of biologically independent mice.

**Extended Data Fig. 1:**
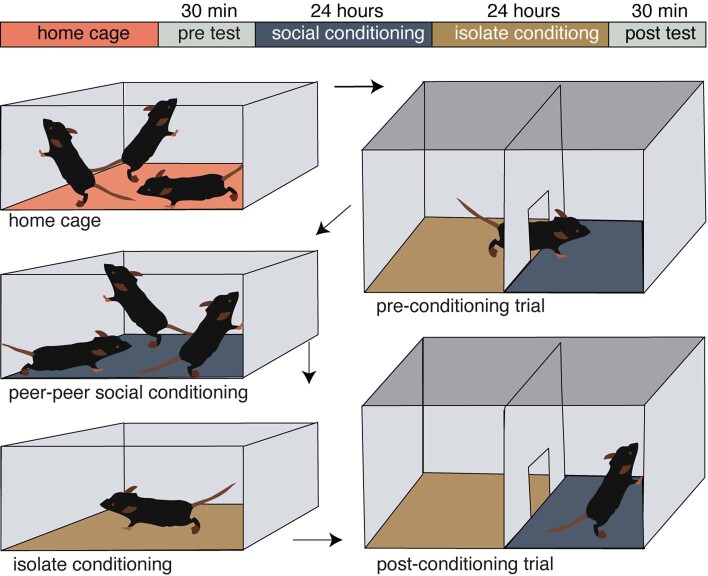
Social conditioned place preference assay. Time course (top) and protocol (bottom) for the social conditioned place preference (social CPP) assay used to measure the reward value of social interactions, where an increased amount of time spent in the bedding paired to the socially conditioned cue in the ‘post’ compared to ‘pre’ trial is interpreted as evidence for a learned association between the positive reward value of social interactions and a novel bedding cue (social reward learning).

**Extended Data Fig. 2:**
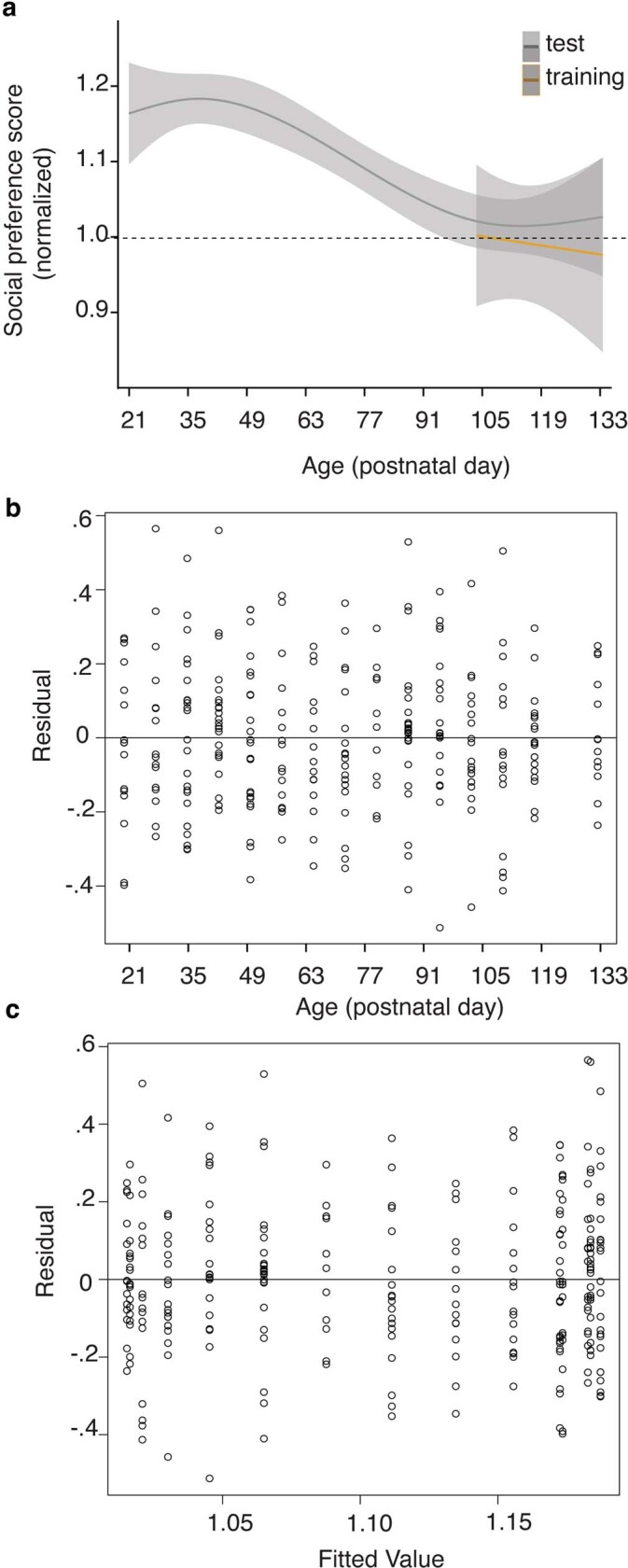
Computation model of social reward critical period. Natural-spline model curve fitting the normalized time spent in social versus isolate cue following social CPP across 15 postnatal ages of not injected (P21-P112) or saline injected (P126) mice from Nardou *et al*. 2019^11^. Ages were chosen to span major milestones in social and brain development including: weaning (postnatal day 21, P21), onset of puberty (P28), sexual maturity (P42), early adulthood (P60), and mature adulthood (P90). (**a**) Plot showing model fit to training and test data indicating a strong concordance. (**b**) Plot of residuals vs age for ns-spline model. (**C**) Plot of residuals vs fitted values for ns-spline model.

**Extended Data Fig. 3:**
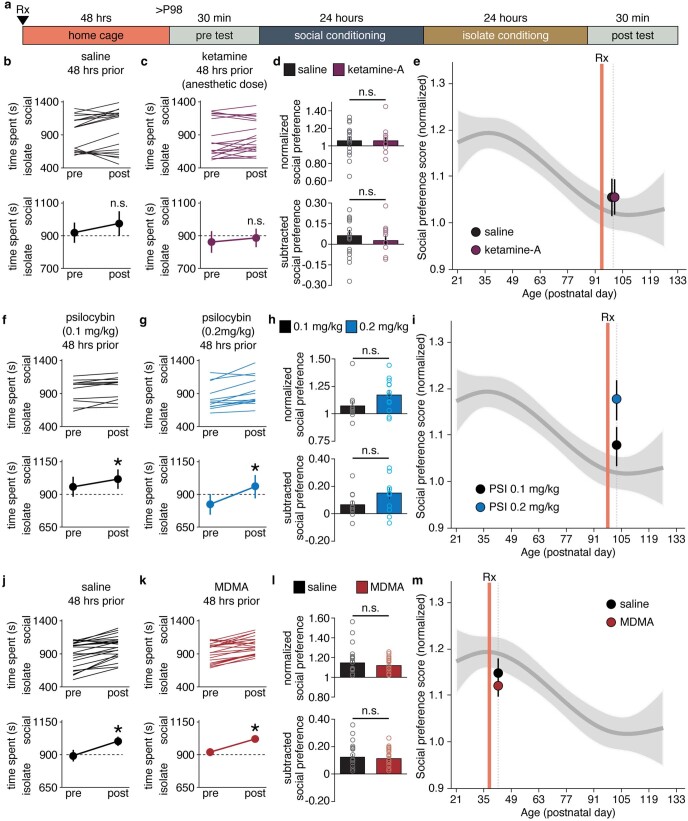
Psychedelic effects are dose, context, and age dependent. (**a**) Diagram illustrating experimental time course of i.p. pretreatment in social CPP. (**b**,**c**) Individual (top) and average (bottom) responses indicate that adult animals do not develop a preference for the social bedding cue after pretreatment with an anesthetic dose of i.p. ketamine, suggesting an important role for context, since social interactions are precluded at this dose (100 mg/kg) (**b**, n = 18, *t*_(17)_ = −1.75, *P* = 0.0986; **c**, n = 20, *t*_(19)_ = −0.89452, P = 0.382). (**d**) Comparisons (two-tailed unpaired *t*-test) reveal no significant difference in normalized (top) and subtracted (bottom) social preference for saline versus ketamine pretreated animals (**d**, normalized, *t*_(36)_ = 0.012694, P = 0.990; subtracted, *t*_(36)_ = −0.7581, *P* = 0.454). (**e**) Normalized social preference of adult animals pretreated with saline and ketamine plotted against ns-spline model of the developmental time course of normalized social preference scores. Comparison to the ns-spine model revealed that the magnitude of sCPP in saline (P = 0.9976) and ketamine (P = 0.9921) pretreated animals did not deviate significantly from the “closed” state. (**f**,**g**) Individual (top) and average (bottom) responses indicate that adult animals develop a significant preference for the social bedding cue after pretreatment with 0.1 mg/kg (**b**, n = 11, *t*_(10)_ = −2.3288, *P* = 0.0421) and 0.2 mg/kg psilocybin (**c**, n = 12, *t*_(11)_ = −3.5499, P = 0.0046). (**h**) Comparisons (two-tailed unpaired *t*-test) reveal no significant difference in normalized (top) and subtracted (bottom) social preference for pretreatment with 0.1 mg/kg versus 0.2 mg/kg psilocybin (**h**, normalized, *t*_(21)_ = −1.6504, *P* = 0.115; subtracted, *t*_(21)_ = −1.7097, *P* = 0.109). (**i**) Normalized social preference of adult animals pretreated with 0.1 mg/kg and 0.2 mg/kg psilocybin plotted against ns-spline model of the developmental time course of normalized social preference scores. Comparison to the ns-spine model revealed that the magnitude of sCPP in animals pretreated with 0.1 mg/kg psilocybin (*P* = 0.7938) did not deviate significantly from the “closed” state, while animals pretreated with 0.2 mg/kg psilocybin (P = 0.04646) demonstrated a significant mean shift in range of the “open” state. (**j**,**k**) Individual (top) and average (bottom) responses indicate that P42 animals develop a significant preference for the social bedding cue after pretreatment with saline (**j**, n = 22, *t*_(21)_ = −5.1882, *P* < 0.001) and MDMA (**k**, n = 22, *t*_(21_) = −5.5432, P < 0.001) (two-tailed paired *t*-test). (**d**), Comparisons (two-tailed unpaired *t*-test) reveal no significant difference in normalized (top) and subtracted (bottom) social preference for pretreatment with MDMA (10 mg/kg) versus saline (**l**, normalized, *t*_(42_) = 0.6877, *P* = 0.495; subtracted, *t*_(42)_ = 0.29151, *P* = 0.772). (**m**) Normalized social preference of P42 animals pretreated with saline and MDMA plotted against ns-spline model of the developmental time course of normalized social preference scores. Comparison to the ns-spine model revealed that the magnitude of sCPP in animals pretreated with 10 mg/kg MDMA (P = 0.1831) or saline (P = .7721) did not deviate significantly from the “open” state; comparisons to spline regression model were considered not significant P > 0.1. Data are presented as mean ± s.e.m. *P < 0.05; n.s., comparisons not significant (P > 0.05). n = X biologically independent animals.

**Extended Data Fig. 4:**
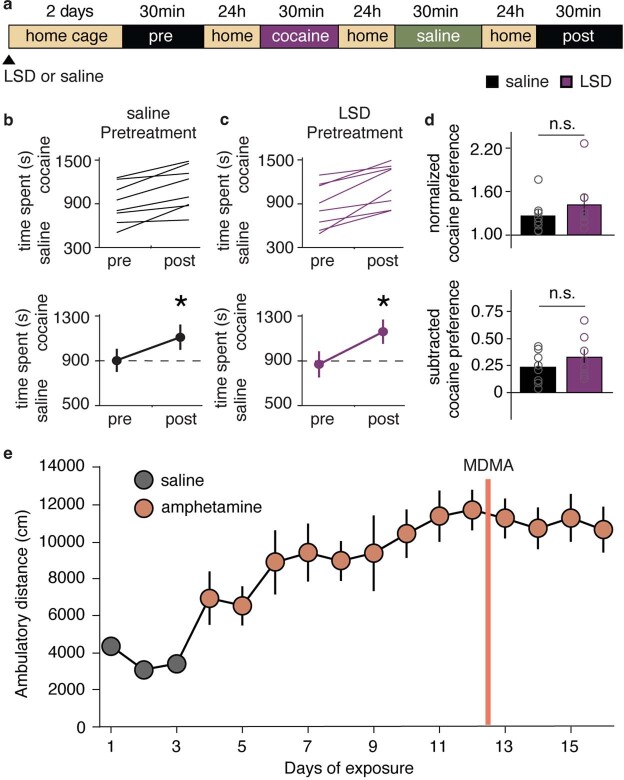
Psychedelic pretreatment does not addiction-like behaviors. (**a**) Diagram illustrating experimental time course of i.p. injections in cocaine CPP. (**b**,**c**) Individual (top) and average (bottom) time spent in the cocaine paired context indicates that mice pretreated with saline (**b**, n = 8, *t*_(7)_ = −4.5102, *P* = 0.0028) and LSD (**c**, n = 8, *t*_(7)_ = −4.8353, *P* = 0.0019) both develop significantly increased preference for the cocaine context after conditioning with 5 mg/kg cocaine (two-tailed paired *t*-test). (**d**) Comparisons reveal no difference in normalized cocaine preference (top, *t*_(14)_ = −1.0051, *P* = 0.332) and subtracted cocaine preference (bottom, *t*_(14)_ = −1.1035, *P* = 0.288) (two-tailed unpaired *t*-test). (**e**) Locomotor activity during sensitization to amphetamine (20 mg/kg) following 3 days of saline pretreatment. Administration of MDMA after day 12 did not significantly alter the locomotor response to amphetamine (day 12 versus day 16, n = 12, *t*_(11)_ = 2.2171, *P* = 0.443). Data are presented as mean ± s.e.m. *P < 0.05; n.s., comparisons not significant (*P* > 0.05). n = X biologically independent animals.

To test whether the ability of MDMA to reopen the social reward learning critical period generalizes across psychedelics, we began by examining the effect of psilocybin pretreatment on the magnitude of social reward learning in adulthood using the social reward conditioned place preference (sCPP) assay (Extended Data Fig. 1). We administered a single intraperitoneal (i.p.) dose of psilocybin^17^ (0.3 mg kg^−1^) to adult male mice (at postnatal day 96 (P96)) and 48 h later (at P98), we assessed the magnitude of sCPP (Fig. 1a). Mice pretreated with psilocybin, but not saline, exhibited a significant sCPP at P98 (Fig. 1b–d). To formally designate ‘open’ and ‘closed’ states of this critical period, we next generated a natural spline regression model to previously published data^11^ with knots at P35 and P98 (*P* = 1.003 × 10^−6^; root mean square error (r.m.s.e.) = 0.19; *R*^2^ = 0.11), as shown in Extended Data Fig. 2. When compared with this derived curve, the magnitude of sCPP in saline-treated mice did not deviate significantly from the closed state (*P* = 0.72), whereas the fit derived from psilocybin-treated mice demonstrated a significant mean shift (*P* = 1.12 × 10^−6^) in range of the open state (Fig. 1e). Similarly, pretreatment with LSD^17^ (i.p. 1 µg kg^−1^) but not saline, also reopened the critical period for social reward learning (saline *P* = 0.90, LSD *P* = 1.76 × 10^−9^) (Fig. 1f–i). Next, we examined the effects of ketamine^18^ (i.p. 3 mg kg^−1^) and ibogaine^19^ (i.p. 40 mg kg^−1^). Mice pretreated with either drug also exhibited sCPP in adulthood (*P* = 8.78 × 10^−4^ and *P* = 3.17 × 10^−5^, respectively) (Fig. 1k–m). As with MDMA^11^, these effects were dose-dependent (Extended Data Fig. 3). In juveniles, MDMA^11^ (i.p. 10 mg kg^−1^) pretreatment did not lead to a further increase the magnitude of social reward learning (Extended Data Fig. 3). In contrast to its effects on social reward learning behaviour, pretreatment with psychedelics had no effect on the magnitude of two addiction-like behaviours: cocaine reward learning and amphetamine-induced locomotor sensitization (Extended Data Fig. 4). Together, these studies demonstrate that as with empathogenic psychedelics^11^, hallucinogenic, oneirogenic and dissociative psychedelics are able to reopen the critical period for social reward learning.

## Duration of the psychedelic open state

**Fig. 2:**
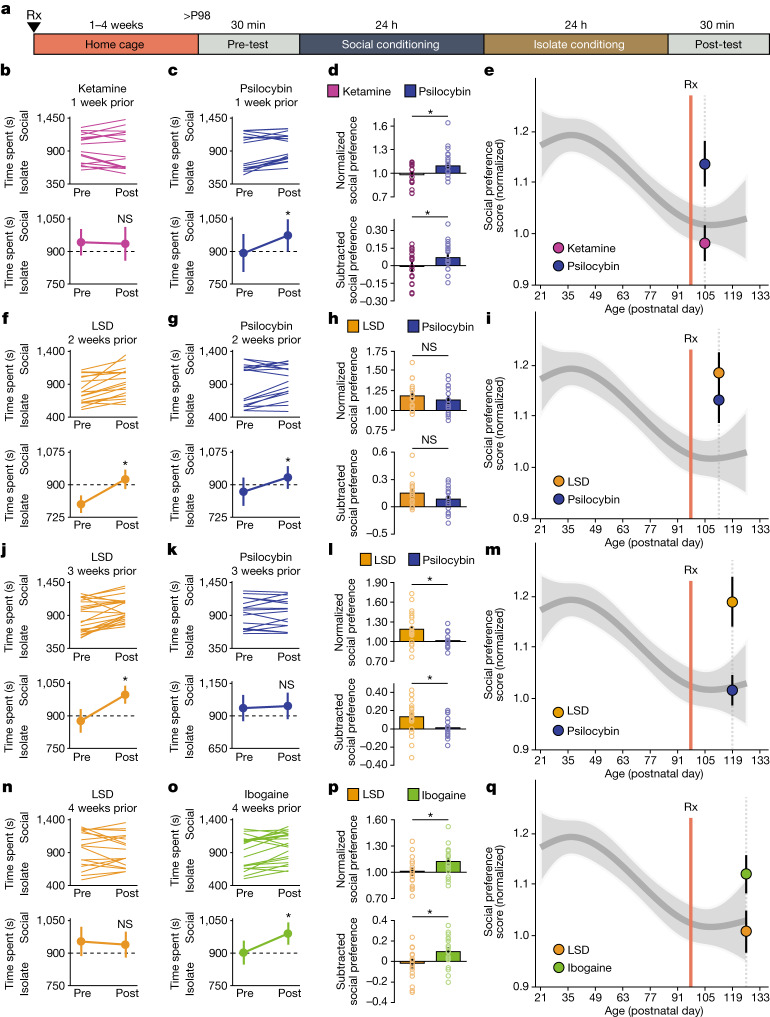
The duration of the open state induced by psychedelics is variable. **a**, Experimental time course of i.p. pretreatment in the sCPP assay. **b**–**q**, sCPP in adult mice 1 week after i.p. pretreatment with ketamine (3 mg kg^−1^) or psilocybin (0.3 mg kg^−1^) (**b**–**e**), 2 weeks after pretreatment with LSD (1 µg kg^−1^) or psilocybin (0.3 mg kg^−1^) (**f**–**i**), 3 weeks after pretreatment with LSD (1 µg kg^−1^) or psilocybin (0.3 mg kg^−1^) (**j**–**m**) or 4 weeks after pretreatment with LSD (1 µg kg^−1^) or ibogaine (40 mg kg^−1^) (**n**–**q**). **b**,**c**,**f**,**g**,**j**,**k**,**n**,**o**, Individual (top) and average (bottom) responses indicate the reinstatement of sCPP is absent one week after ketamine treatment (**b**, *n* = 16 mice, *t*_(15)_ = 0.204, *P* = 0.841), lasts two weeks for psilocybin (**c**, 1 week: *n* = 17 mice, *t*_(16)_ = −2.959, *P* = 0.009; **g**, 2 weeks: *n* = 22 mice, *t*_(21)_ = −3.542, *P* = 0.002; **k**, 3 weeks: *n* = 16 mice, *t*_(15)_ = −0.405, *P* = 0.691), lasts 3 weeks for LSD (**f**, 2 weeks: *n* = 18 mice, *t*_(17)_ = −4.360, *P* < 0.001; **j**, 3 weeks: *n* = 23 mice, *t*_(22)_ = −3.671, *P* = 0.001; **n**, 4 weeks: *n* = 17 mice, *t*_(16)_ = 0.441, *P* = 0.665), and lasts at least 4 weeks for ibogaine (**o**, *n* = 20 mice, *t*_(19)_ = −3.004, *P* = 0.007). Two-tailed paired *t*-test. **d**,**h**,**l**,**p**, Comparisons reveal a significant difference in sCPP between ketamine and psilocybin groups 1 week after pretreatment (**d**, normalized: *t*_(31)_ = −2.700, *P* = 0.011; subtracted: *t*_(31)_ = −2.113, *P* = 0.043), between LSD and psilocybin at 3 weeks (**l**, normalized: *t*_(34)_ = 3.050, *P* = 0.004; subtracted: *t*_(37)_ = 2.471, *P* = 0.018) but not at 2 weeks (**h**, normalized: *t*_(38)_ = 0.390, *P* = 0.699; subtracted: *t*_(38)_ = 1.077, *P* = 0.288), and LSD and ibogaine 4 weeks after pretreatment (**p**, normalized: *t*_(35)_ = −2.045, *P* = 0.048; subtracted: *t*_(35)_ = −2.283, *P* = 0.029). Two-tailed unpaired *t*-test, with Welch’s correction to account for unequal variance in **l** subtracted. *P < 0.05; NS, not significant (*P* > 0.05). **e**,**i**,**m**,**q**, Normalized social preference one week after ketamine or psilocybin (**e**), two (**i**) and three (**m**) weeks after LSD and psilocybin, and four weeks after LSD and ibogaine (**q**) plotted against a natural spline model of the developmental time course of normalized social preference scores. The magnitude of sCPP did not deviate significantly from the closed state 1 week after ketamine (**e**, *P* = 0.949), three weeks after psilocybin (**i**, *P* = 0.633) and four weeks after LSD (**m**, *P* = 0.705), whereas the magnitude demonstrated a significant mean shift in range of the open state for both one (**e**, *P* = 0.054) and two weeks (**i**, *P* = 0.0211) after psilocybin, two (**i**, *P* = 0.0121) and three weeks (**m**, *P* = 0.00745) after LSD and four weeks after ibogaine (**q**, *P* = 0.0758). Comparisons to the natural spline model were considered not significant (*P* > 0.1). Data are mean ± s.e.m. *n* refers to the number of biologically independent mice.

**Fig. 3:**
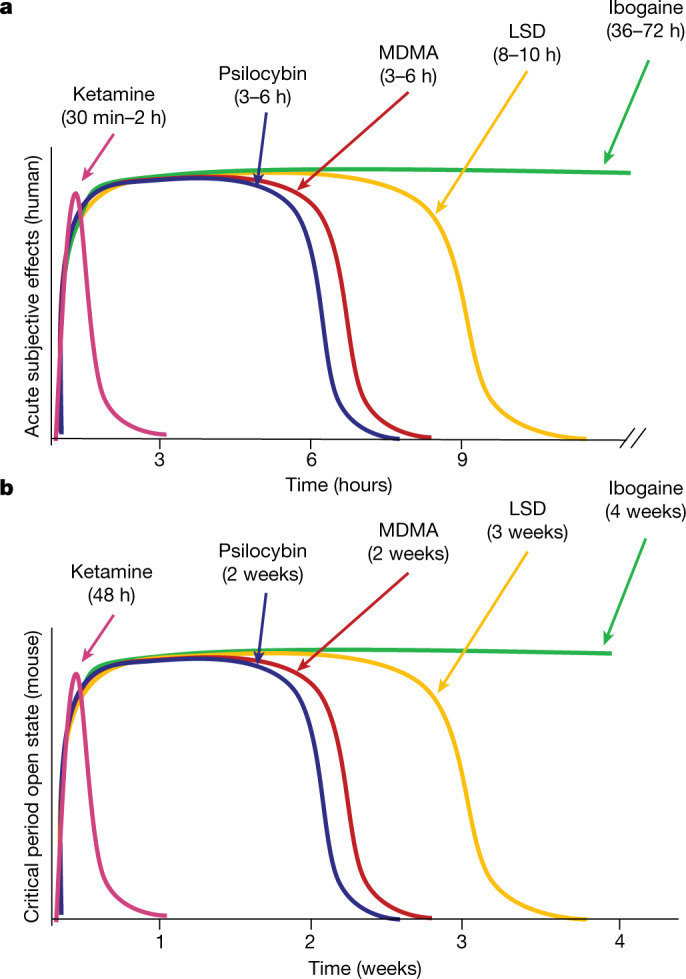
The durations of acute subjective effects in humans are proportional to the durations of the critical period open state in mice. **a**, Durations of the acute subjective effects of psychedelics in humans (data from refs. ^15,16,20–22^). **b**, Durations of the critical period open state induced by psychedelics in mice. Based on ref. ^11^ and Figs. 1 and 2 and Extended Data Fig. 5.

**Extended Data Fig. 5:**
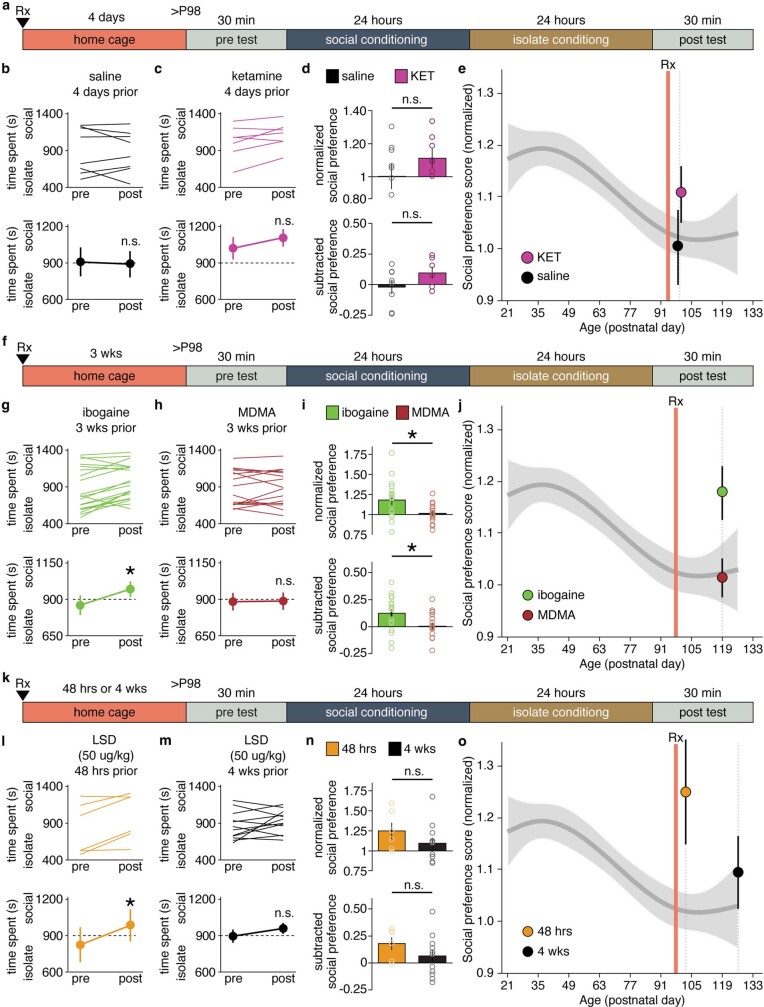
Duration of the open state induced by psychedelics is variable but not extended by increasing dose. (**a**,**f**,**k**) Diagrams illustrating experimental time course of i.p. pretreatment in social CPP. (**b**,**c**) Individual (top) and average (bottom) responses indicate that adult animals do not develop a significant preference for the social bedding cue 4 days after pretreatment with saline (**b**, n = 8, *t*_(7)_ = 0.38628, *P* = 0.711) and ketamine (3 mg/kg) (**c**, n = 7, *t*_(6)_ = −2.2235, *P* = 0.068) (two-tailed paired *t*-test). (**d**) Comparisons (two-tailed unpaired *t*-test) reveal no significant difference in normalized (top) and subtracted (bottom) social preference for 4 days after pretreatment with ketamine versus saline (**d**, normalized, *t*_(13)_ = t = −1.2142, *P* = 0.258; subtracted, *t*_(13)_ = −1.6823, *P* = 0.123). (**e**) Comparison to the ns-spine model revealed that the magnitude of sCPP did not deviate significantly from the “closed” state four days after saline (P = 0.508) and ketamine (P = 0.4418). (**g**,**h**) Individual (top) and average (bottom) responses indicate the reinstatement of social CPP is present three weeks after pretreatment with ibogaine (**g**, 40 mg/kg, n = 20, *t*_(19)_ = −3.5381, *P* = 0.0022), but absent with MDMA (**h**, 10 mg/kg, n = 16, t_(15)_ = −0.11467, *P* = 0.968) (two-tailed paired *t*-test). (**i**) Comparisons (two-tailed unpaired *t*-test) reveal a significant difference in normalized (top, *t*_(34)_ = 2.615, P = 0.0170) and subtracted (bottom, t(34) = 2.4756, P = 0.0204) social preference 3 weeks after pretreatment with ibogaine versus MDMA. Comparison to the ns-spine model revealed that the magnitude of sCPP demonstrated a significant mean shift in range of the “open” state for 3 weeks after ibogaine (P = 0.01158), while the magnitude did not deviate significantly from the “closed” state for 3 weeks after MDMA (P = 0.9152). (**k**,**l**) Individual (top) and average (bottom) responses indicate that the reinstatement of social CPP is present 48 h after pretreatment with LSD (**k**, n = 6, *t*_(5)_ = −3.0103, *P* = 0.0297), but absent 4 weeks after (**l**, n = 12, *t*_(11)_ = −1.1834, *P* = 0.262). (**m**) Comparisons reveal non-significant difference in normalized (top) and subtracted (bottom) social preference for 48 h versus 4 weeks after pretreatment with LSD (**m**, normalized, *t*_(16)_ = 1.2786, P = 0.2314; subtracted, *t*_(16)_ = 1.4209, P = 0.1792) (two-tailed unpaired t-test). (**n**) Comparison to the ns-spine model revealed that the magnitude of sCPP demonstrated a significant deviation to the “closed” state at 48 h (P = .0915), while the magnitude did not deviate significantly from the “closed” state at 4 weeks (P = 0.3168); comparisons to spline regression model were considered not significant P > 0.1. Data are presented as mean ± s.e.m. *P < 0.05; n.s., comparisons not significant (P > 0.05). n = X biologically independent animals.

The duration of acute subjective effects and the durability of the therapeutic response vary considerably across psychedelics. For example, in humans, the acute subjective effects of ketamine^15^ last 30–120 min, whereas its antidepressant effects^9^ last for 1 week. By contrast, the subjective effects of psilocybin and MDMA^20,21^ last for 3–6 h, whereas the acute effects of LSD and ibogaine persist for 8–10 h and 36–72 h, respectively^16,22^; these long-lasting subjective effects correspond to highly durable therapeutic effects that last months to years^4,5,7^. Previously, we showed that MDMA-induced critical period reopening lasts for two weeks, but returns to the closed state by four weeks^11^. Here, to further probe the time course of the critical period open state induced by psychedelics, we examined the duration of critical period reopening following treatment with ketamine, psilocybin, LSD and ibogaine (Fig. 2a). One week following psychedelic treatment, psilocybin-treated mice, but not those treated with ketamine, exhibited significant social reward learning (Fig. 2b–e). Two weeks following psychedelic treatment, the social reward learning critical period remained open for both psilocybin- and LSD-treated mice (Fig. 2f–i). At three weeks, LSD-treated mice, but not those treated with psilocybin, exhibited significant social reward learning (Fig. 2j–m), whereas at four weeks, the social reward learning critical period remained open for mice treated with ibogaine but not those treated with LSD (Fig. 2n–q). For each psychedelic, we examined at least three time points; increasing the LSD dose to 50 µg kg^−1^ did not extend the duration of the open state (Extended Data Fig. 5). As shown in Fig. 3, the progressively longer-lasting open states induced by ketamine (Figs. 1f–i and 2b–e and Extended Data Fig. 5), followed by psilocybin (Fig. 2b–i), MDMA^11^ (Extended Data Fig. 5), LSD (Fig. 2j–q) and ibogaine (Fig. 2n–q and Extended Data Fig. 5) are proportional to the duration of the acute subjective effects of these drugs in humans^15,16,20–22^. These results provide a mechanistic explanation for the importance of the post-treatment integration period for clinical implementation of psychedelics, and inform the design of novel compounds for clinical applications.

## Metaplasticity, not hyperplasticity

**Fig. 4:**
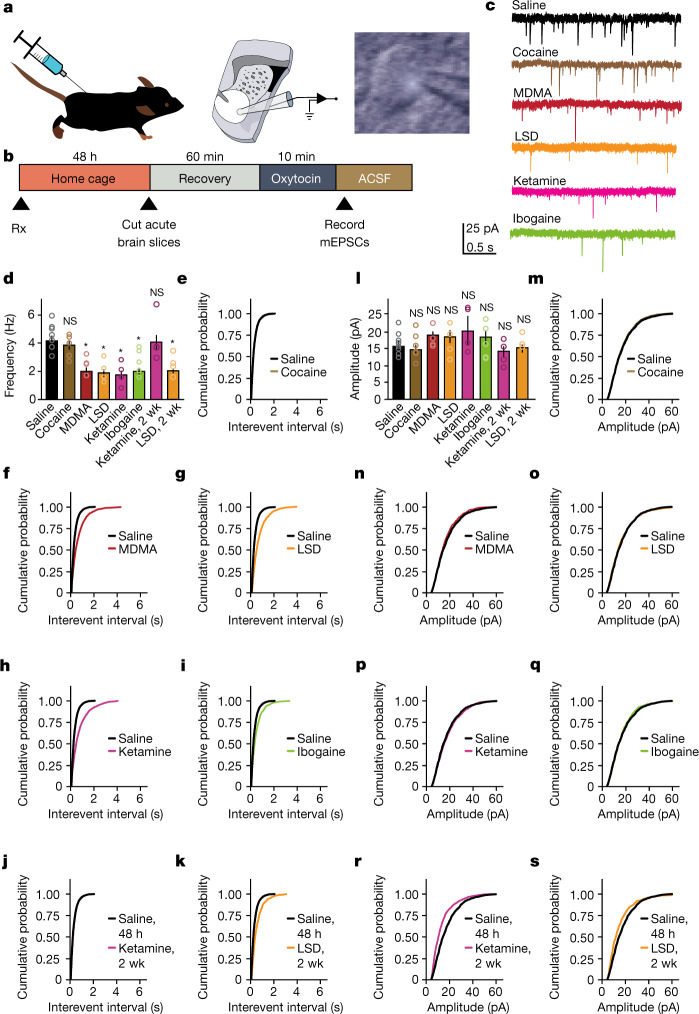
Psychedelics induce metaplasticity. **a**,**b**, Illustration (**a**) and time course (**b**) of treatment and electrophysiology protocol. Illustration in **a** adapted from ref. ^25^. **c**, Representative mEPSC traces recorded from MSNs in the NAc of oxytocin-treated brain slices collected from mice pretreated with saline (*n* = 8), 20 mg kg^−1^ cocaine (*n* = 6), 10 mg kg^−1^ MDMA (*n* = 4), 1 µg kg^−1^ LSD (*n* = 4), 3 mg kg^−1^ ketamine (*n* = 4) or 40 mg kg^−1^ ibogaine (*n* = 5). **d**–**k**, Average frequency of mEPSCs (**d**) and cumulative probabilities of interevent intervals for cocaine (**e**), MDMA (**f**), LSD (**g**), ketamine (**h**) and ibogaine (**i**) recorded from MSNs after two days, and after two weeks (wk) for ketamine (**j**) and LSD (**k**). **l**–**s**, Average (**l**) and cumulative probability distributions of amplitudes recorded from MSNs for cocaine (**m**), MDMA (**n**), LSD (**o**), ketamine (**p**) and ibogaine (**q**) recorded from MSNs after two days, and after two weeks for ketamine (**r**) and LSD (**s**). One-way analysis of variance revealed a significant effect of treatment on frequency (**d**, *F*_(7,31)_ = 5.99, *P* = 0.0002) but not amplitude (**l**, *F*_(7,31)_ = 1.09, *P* = 0.39), and multiple comparison analysis revealed an oxytocin-mediated decrease in mEPSC frequency after pretreatment with psychedelics (**f**, MDMA: *P* = 0.011; **g**, LSD: *P* = 0.0013; **h**, ketamine: *P* = 0.001; **i**, ibogaine: *P* = 0.013), but not cocaine (*P* = 0.83), and that this decrease remained significant at the two-week time point with LSD (**k**, *n* = 4, *P* = 0.01) but not ketamine (**j**, *n* = 4, *P* = 0.99). All cells have been recorded in slices of adult mice at P98. Data are mean ± s.e.m. *P < 0.05; NS, not significant (*P* > 0.05). *n* refers to the number of biologically independent cells.

**Extended Data Fig. 6:**
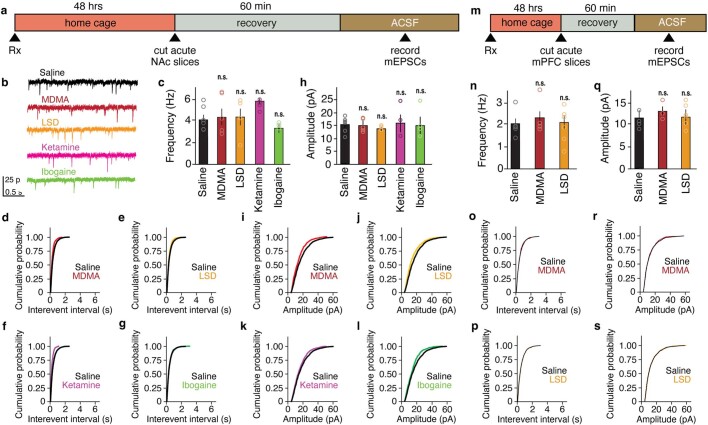
Psychedelics do not induce hyperplasticity in the NAc or mPFC. (**a**) Time course of treatment and electrophysiology protocol. (**b**) Representative mEPSC traces recorded from MSNs in the NAc of brain slices collected from mice pretreated 48 previous with saline (n = 6), 10 mg/kg MDMA (n = 6), 1 µg/kg LSD (n = 4), 3 mg/kg ketamine (n = 4), and 40 mg/kg ibogaine (n = 4). (**c**) Average frequency of mEPSCs and (**d**–**g**) cumulative probabilities of inter event intervals recorded from MSNs. (**h**) Average and (**i**–**l**) cumulative probability distributions of amplitudes recorded from MSNs. One-way analysis of variance revealed no significant effect of treatment on frequency (F_(5,21)_ = 1.05, P = 0.41) or amplitude (F_(5,21)_ = 0.16, P = 0.97). (**m**) Time course of treatment and electrophysiology protocol for mEPSCs recorded in layer 5 pyramidal neurons in the mPFC of brain slices collected from mice pretreated 48 previous with saline (n = 5), 10 mg/kg MDMA (n = 4), 1 µg/kg LSD (n = 6). (**n**) Average frequency of mEPSCs and (**o**–**p**) cumulative probabilities of inter event intervals recorded from layer 5 pyramidal neurons. (**q**) Average and (**r**–**s**) cumulative probability distributions of amplitudes recorded from layer 5 pyramidal neurons. One-way analysis of variance revealed no significant effect of treatment on frequency (F_(2,12)_ = 0.34, P = 0.72) or amplitude (F_(2,12)_ = 0.26, P = 0.78). All cells have been recorded in slices of adult animals at P98. Data are presented as mean ± s.e.m. *P < 0.05, n.s. comparisons not significant P > 0.05. n = X biologically independent cells.

Dynamic regulation of the extent to which synaptic plasticity can be induced is called ‘metaplasticity’^23^, and is thought to be one of the mechanisms underlying the establishment of critical periods^24^. Previously, we showed that oxytocin induces a novel form of presynaptically expressed long-term depression, and implicated this plasticity in encoding social reward learning^25,26^. Here, to determine whether the ability to induce metaplastic upregulation of oxytocin plasticity generalizes across psychedelics, we pretreated adult mice with either saline, cocaine or psychedelics. Forty-eight hours or two weeks later we prepared ex vivo acute slices containing the nucleus accumbens (NAc) and conducted whole-cell voltage-clamp recordings from medium spiny neurons (MSNs) (Fig. 4a–c). A 10-min bath application of oxytocin induced a significant decrease in the frequency (Fig. 4d–k) but not the amplitude (Fig. 4l–s) of miniature excitatory post-synaptic currents (mEPSCs) following pretreatment with MDMA, LSD, psilocybin, ketamine and ibogaine, but not with saline or cocaine, at 48 h; this metaplasticity persisted for 2 weeks in the LSD pretreatment group, but not in the ketamine pretreatment groups. We did not observe significant changes in baseline mEPSC amplitude or frequency following pretreatment with psychedelics in the NAc or in layer 5 of the medial prefrontal cortex (mPFC) (Extended Data Fig. 6). Together, these results provide evidence that psychedelics induce metaplasticity rather than hyperplasticity, a distinction that is especially important for designing biomarkers to test therapeutic profiles and abuse liability of novel compounds.

## 5-HT_2A_R is not the universal mechanism

**Extended Data Fig. 7:**
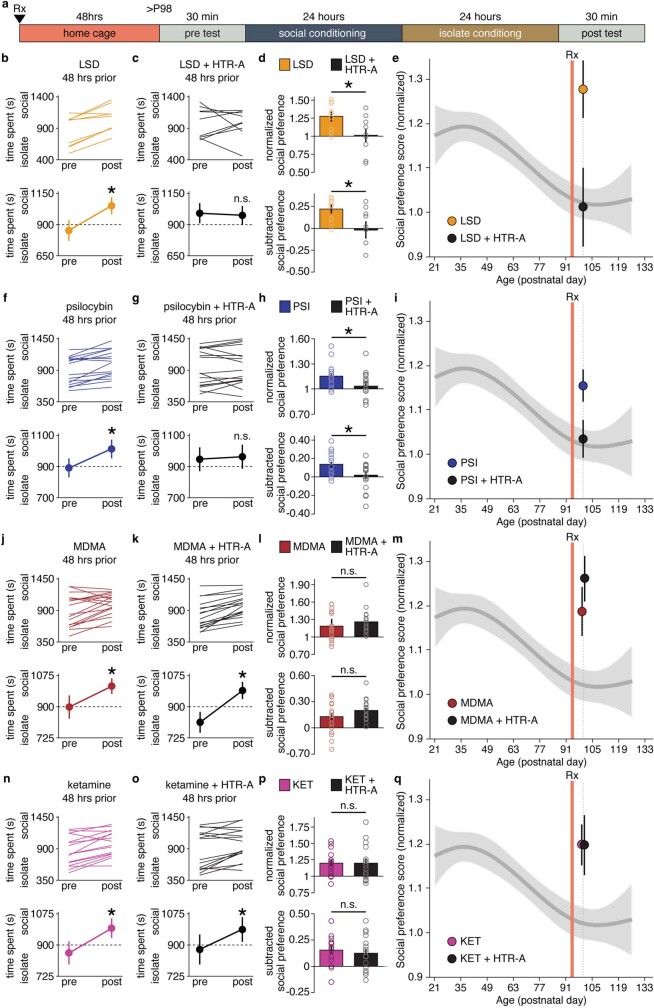
Serotonin 2A receptors are not universally required for critical period reopening. (**a**) Diagram illustrating experimental time course of i.p. pretreatment in social CPP. (**b**,**c**,**f**,**g**,**j**,**k**,**n**,**o**) Individual (top) and average (bottom) responses of P98 animals indicate that ketanserin (HTR-A) abolished the reopening of social reward learning critical period by LSD (**b**, LSD, n = 9 animals, t_(8)_ = −4.938, P = 0.001; **c**, LSD + HTR-A, n = 9 animals, t_(8)_ = 0.210, P = 0.839), and psilocybin (**f**, psilocybin n = 16 animals, t_(15)_ = −4.494, P < 0.001; **g**, psilocybin + HTR-A, n = 17 animals, t_(16)_ = −0.515, P = 0.613), but not by MDMA (**j**, MDMA, n = 18 animals, t_(17)_ = −2.916, P = 0.01 ; **k**, MDMA + HTR-A, n = 17 animals, t_(16)_ = −6.737, P < 0.001) and ketamine (**n**, ketamine n = 16 animals, t_(15)_ = −4.517, P < 0.001; **o**, ketamine + HTR-A, n = 16 animals, t_(15)_ = −2.952, P < 0.001) (two tailed paired t-test). (**d**,**h**,**l**,**p**) Comparisons of the normalized (top) and subtracted (bottom) social preference between treatment groups reveal a decrease following LSD + HTR-A versus LSD alone (**d**, normalized, t_(16)_ = 2.427, P = 0.027; subtracted, t_(16)_ = 2.377, P = 0.030), a decrease following psilocybin + HTR-A vs psilocybin alone (**h**, normalized, t_(31)_ = 2.114, P = 0.043; subtracted, t_(31)_ = 2.475, P = 0.019), but no difference between MDMA and MDMA + HTR-A pretreatment groups (**l**, normalized, t_(33)_ = −0.971, P = 0.339; subtracted, t_(33)_ = −1.282, P = 0.209), nor ketamine and ketamine + HTR-A pretreatment groups (**p**, normalized, t_(30)_ = 0.013, P = 0.990; subtracted, t_(30)_ = 0.535, P = 0.597) (two tailed unpaired t-test). (**e**,**i**,**m**,**q**) Normalized social preference in mice pretreated with LSD (**e**), psilocybin (**i**), MDMA (**m**) and ketamine (**q**) in the presence or the absence of HTR-A plotted against ns-spline model of the developmental time course of normalized social preference scores. Comparison to the ns-spine model revealed that the magnitude of sCPP did not deviate significantly from the “closed” state for LSD + HTR-A (P = 0.728), and psilocybin + HTR-A (P = 0.987), while the magnitude demonstrated a significant mean shift in range of the “open” state for LSD (P = 2.52e-09), psilocybin (P = 1.43e-06), MDMA in presence (P = 1.77e-05) or absence of HTR-A (P = 0.22e-4), and ketamine in presence (P = 0.002619) or absence of HTR-A (P = 0.000996); comparisons to spline regression model were considered not significant P > 0.1. Data are presented as mean ± s.e.m. *P < 0.05, n.s. comparisons not significant P > 0.05. n = X biologically independent animals.

The serotonin receptor 5-HT_2A_R, first identified by its binding to LSD^27^, mediates alterations of perception and cognition induced by ‘serotonergic psychedelics’^10^ such as LSD^28^ and psilocybin^29^. Furthermore, MDMA is thought to trigger synaptic efflux of serotonin through its binding at the serotonin transporter SERT^30^, and some of the effects of ketamine are reportedly mediated by 5-HT_2A_R^31^. Thus, we sought to determine the role of 5-HT_2A_R in reopening the social reward learning critical period with LSD, psilocybin, MDMA and ketamine. We administered psychedelics intraperitoneally in P96 adult mice either alone or in combination with ketanserin (HTR-A, 0.1 mg kg^−1^)—the 5-HT_2A_R antagonist used in human studies—which we injected 30 min before the psychedelic (Extended Data Fig. 7). Pre-treatment with either LSD or psilocybin induced reinstatement of sCPP measured 48 h later, and this effect was blocked by co-administration of ketanserin (Extended Data Fig. 7). However, MDMA-induced reinstatement of sCPP persisted in the presence of ketanserin (Extended Data Fig. 7). Similarly, co-administration of ketanserin did not block ketamine-induced reinstatement of social reward learning in adulthood (Extended Data Fig. 7). These results demonstrate that whereas 5-HT_2A_Rs are required for LSD- and psilocybin-induced reopening of the social reward learning critical period (with potential contributions from serotonin 2B and 2C receptors, since ketanserin also has affinity at these serotonin receptor 2 subtypes), MDMA and ketamine reinstate social reward learning in a 5-HT_2A_R-independent manner. Although some have argued^10,32^ that psychedelics that bind 5-HT_2A_R (such as LSD and psilocybin) should be classified separately from those that do not (such as MDMA and ketamine), these results identify a novel property (critical period reopening) that coheres the category of psychedelics but violates the 5-HT_2A_R-binding boundary. Thus, combined with the data presented in Figs. 1 and 2, these results support the continued use of the established naming convention for psychedelics^1,2^, rather than subclassification or renaming based on receptor binding or subjective properties.

## β-arrestin-2 is not the universal mechanism

**Extended Data Fig. 8:**
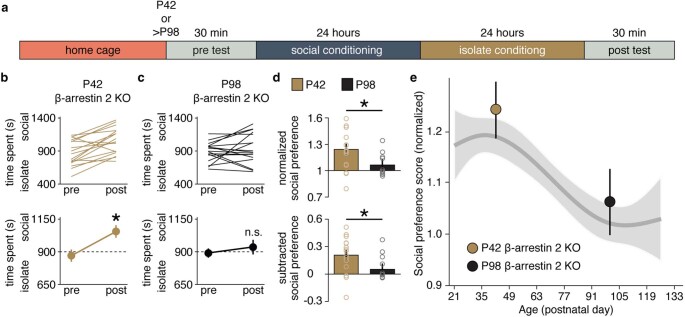
β-arrestin 2 KO mice exhibit normal maturational profile of social reward learning. (**a**) Diagram illustrating experimental time course of social CPP. (**b**–**e**) Social CPP in juvenile (P42) and adult (P98) β-arrestin 2 KO mice. (**b**,**c**) Individual (top) and average (bottom) responses of adult animals indicate that only juvenile β-arrestin 2 KO mice develop a preference for the social bedding cue (P42 (n = 17 animals, t_(16)_ = −4.392, P < 0.001), P98 (n = 17 animals, t_(16)_ = −0.922, P = 0.370) (two tailed paired t-test)). (**d**) Comparisons reveal a difference in normalized (top, t_(32)_ = 2.110, P = 0.043) and subtracted (bottom, t_(32)_ = 2.120, P = 0.042) social preference at P42 versus P98 mice (two tailed unpaired t-test). (**e**) Normalized social preference of β-arrestin 2 KO mice at P42 and P98 plotted against ns-spline model of the developmental time course of normalized social preference scores. Comparison to the ns-spine model revealed that the magnitude of sCPP did not deviate significantly from the “open” state at P42 (P = 0.840) and from the “closed” state at P98 (P = 0.760); comparisons to spline regression model were considered not significant P > 0.1. Data are presented as mean ± s.e.m. *P < 0.05, n.s. comparisons not significant P > 0.05. n = X biologically independent animals.

**Extended Data Fig. 9:**
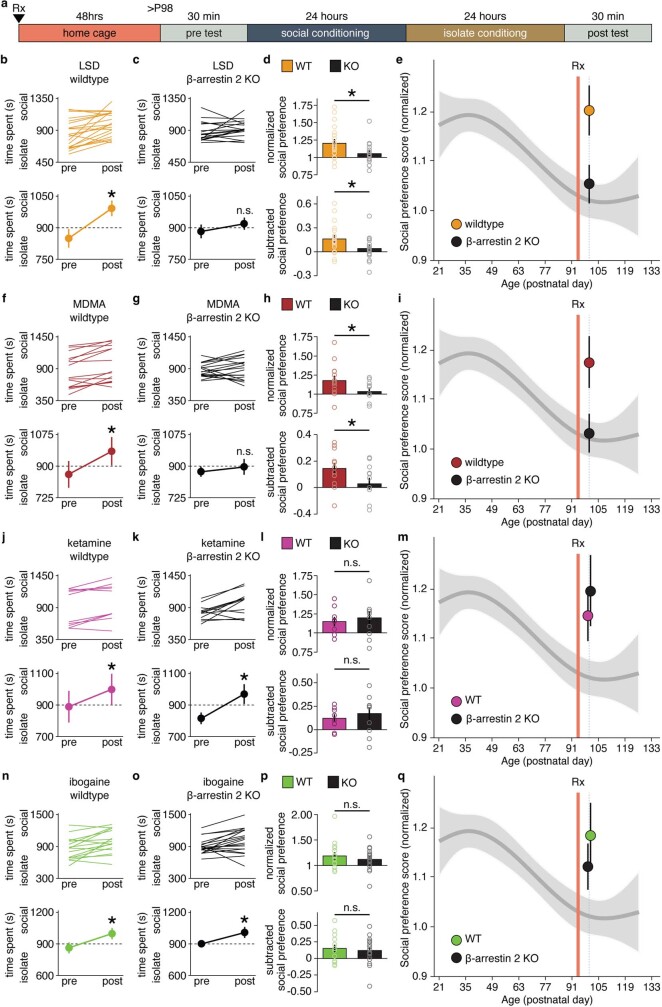
β-arrestin 2 is not universally required for critical period reopening. (**a**) Diagram illustrating experimental time course of i.p. pretreatment in social CPP. (**b**,**c**,**f**,**g**,**j**,**k**,**n**,**o**) Individual (top) and average (bottom) responses of P98 animals indicate that LSD (**b**, WT, n = 21 animals, t_(20)_ = −3.992, P < 0.001; **c**, β-arrestin 2 KO, n = 17 animals, t_(16)_ = −1.105, P = 0.286) and MDMA (**f**, WT, n = 15 animals, t_(14)_ = −3.994, P = 0.001; **g**, β-arrestin 2 KO, n = 18 animals, t_(17)_ = −0.759, P = 0.458) don’t reopen the social reward learning critical period in β-arrestin 2 KO mice whereas ketamine (**j**, WT, n = 10 animals, t_(9)_ = −3.448, P = 0.007; **k**, β-arrestin 2 KO, n = 12 animals, t_(11)_ = −2.903, P = 0.014) and ibogaine (**n**, WT, n = 16 animals, t_(15)_ = −3.135, P = 0.007; **o**, β-arrestin 2 KO, n = 18 animals, t_(17)_ = −2.655, P = 0.016) do reopen the critical period both in WT and in β-arrestin 2 KO mice (two tailed paired t-test). (**d**,**h**,**l**,**p**) Comparisons of the normalized (top) and subtracted (bottom) social preference in WT and β-arrestin 2 KO mice reveal a difference in the magnitude of sCPP after pretreatment with LSD (**d**, normalized, t_(36)_ = 2.248, P = 0.031; subtracted, t_(36)_ = 2.139, P = 0.039), and MDMA (**h**, normalized, t_(31)_ = 2.227, P = 0.033; subtracted, t_(31)_ = 2.112, P = 0.043), but no difference after ketamine (**l**, normalized (t_(20)_ = −0.545, P = 0.591, subtracted, t_(20)_ = −0.676, P = 0.507) and ibogaine (**p**, normalized, t_(33)_ = 0.790, P = 0.435; subtracted, t_(33)_ = 0.462, P = 0.647) (two tailed unpaired t-test). (**e**,**i**,**m**,**q**) Normalized social preference in WT and β-arrestin 2 KO mice pretreated with LSD (**e**), MDMA (**i**), ketamine (**l**), and ibogaine (**q**) plotted against ns-spline model of the developmental time course of normalized social preference scores of male mice. Comparison to the ns-spine model revealed that the magnitude of sCPP did not deviate significantly from the “closed” state in β-arrestin 2 KO mice for LSD (P = 0.357) and MDMA (P = 0.490), while the magnitude demonstrated a significant mean shift in range of the “open” state for WT pretreated with LSD (WT, P = .00315), or MDMA (WT, P = .0140), and for WT and KO pretreated with ketamine (WT, P = .00911; β-arrestin 2 KO, P = .0198) or ibogaine (WT, P = .0249; β-arrestin 2 KO, P = .0954); comparisons to spline regression model were considered not significant P > 0.1. Data are presented as mean ± s.e.m. *P < 0.05, n.s. comparisons not significant P > 0.05. n = X biologically independent animals.

Recent studies indicate that prolonged binding at the 5-HT_2A_R by LSD triggers β-arrestin-2 (β-arr2)-biased signalling over canonical G-protein signalling^33^. Moreover, the effects of MDMA^11^ and ibogaine^34^ are also thought to be mediated by metabotropic G-protein-coupled receptors (GPCRs). Although the therapeutic effects of ketamine are thought to be mediated by ionotropic NMDA receptors^35^, the metabotropic glutamate receptor 5 has also been implicated^36^. To test the hypothesis that β-arr2-biased signalling mediates the ability of psychedelics to reopen the social reward learning critical period, we examined their effects in commercially available β-arr2-knockout (KO) mice. We began by determining baseline sCPP in juvenile and adult β-arr2-KO mice and found that these mice exhibited the normal maturational profile of social reward learning (Extended Data Fig. 8). Next, we compared the magnitude of sCPP in adult (P98) β-arr2 wild-type and β-arr2-KO mice 48 h following administration of psychedelic drugs (Extended Data Fig. 9). LSD and MDMA reopened the social reward learning critical period in wild-type mice but did not do so in β-arr2-KO mice (Extended Data Fig. 9). Conversely, ketamine and ibogaine were able to reinstate social reward learning in both wild-type and β-arr2-KO mice (Extended Data Fig. 9). Together, these results demonstrate that whereas β-arr2 signalling is required for LSD- or MDMA-induced reopening of the social reward learning critical period, ketamine or ibogaine reinstate social reward learning in a β-arr2-independent manner.

## Psychedelics induce remodelling of the ECM

Since psychedelics as a class all reopen the social reward learning critical period (Fig. 1) even though these drugs act on a diverse array of principal binding targets (Extended Data Fig. 7) and biochemical signalling pathways (Extended Data Fig. 9), we reasoned that the common mechanism that enables critical period reopening might be downstream of these cellular processes. Furthermore, given the durability of the response (Fig. 2), we hypothesized that psychedelics may modulate the expression of specific genes or pathways. To test this hypothesis, we carried out RNA sequencing of the microdissected NAc 48 h and 2 weeks after pretreatment with either saline, cocaine, ketamine, LSD or MDMA. We collected total mRNA from each sample and made strand-specific libraries for each of three replicates from each condition. Transcript-level abundances were collapsed to gene-level expression estimates for model fitting.

**Fig. 5:**
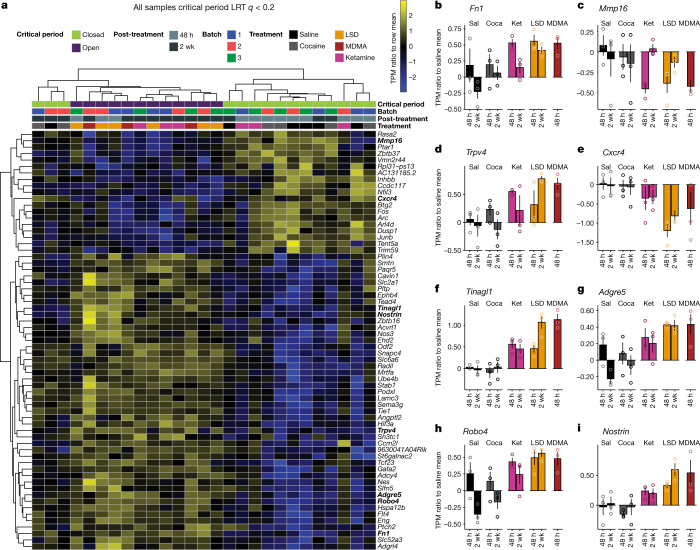
Characteristic changes in transcription induced by psychedelics. **a**, Heat map of normalized RNA expression values from the microdissected NAc for genes that are significantly differentially expressed in conditions where the critical period remains in the open state versus conditions where the critical period remains in or returns to the closed state. LRT, likelihood ratio test; TPM, transcripts per million. **b**–**i**, Ratio of expression values to average saline baseline for top scoring genes related to extracellular matrix remodelling: *Fn1* (**b**), *Mmp16* (**c**), *Trpv4* (**d**), *Cxcr4* (**e**), *Tinagl1* (**f**), *Adgre5* (**g**), *Robo4* (**h**) and *Nostrin* (**i**). Coca, cocaine; ket, ketamine; sal, saline.

**Fig. 6:**
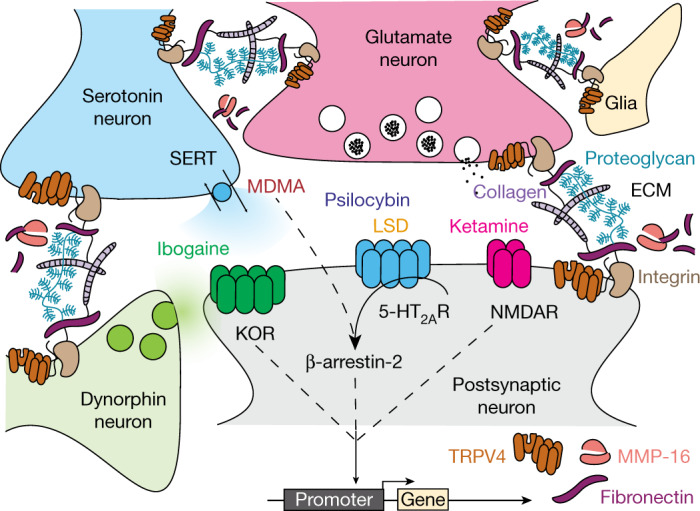
Working model of convergent cellular mechanisms of psychedelics. Psychedelics act on a diverse array of principal binding targets and downstream signalling mechanisms that are not limited to the serotonin 2A receptor (Extended Data Fig. 7) or β-arr2 (Extended Data Fig. 9). Instead, mechanistic convergence occurs at the level of DNA transcription (Fig. 5). Dynamically regulated transcripts include components of the extracellular matrix (ECM) such as fibronectin, as well as receptors (such as TRPV4) and proteases (such as MMP-16) implicated in regulating the ECM. Adapted from ref. ^25^.

**Extended Data Fig. 10:**
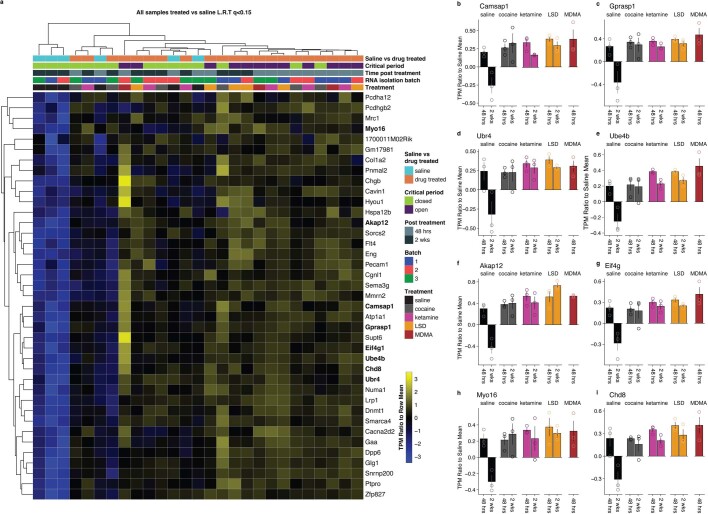
Comparison of gene expression for saline versus all psychoactive drug (including cocaine) treatment groups. (**a**) Heatmap of normalized RNA expression values from the microdissected NAc for genes significantly differentially expressed between treatment with any psychoactive drug including cocaine vs. the saline control. (**b**–**i**) Ratio of expression values to average saline baseline for top scoring genes from this analysis related to increased synaptic transmission.

To directly compare treatment-related transcriptional changes specific to the shared ability of psychedelics to reopen the social reward learning critical period, we analysed the gene expression dataset between conditions in which the critical period is in the open state (48 h and 2 weeks after LSD treatment, 48 h after ketamine treatment, and 48 h after MDMA treatment) versus conditions where the critical period remains in or returns to the closed state (48 h and two weeks after saline treatment, 48 h and two weeks after cocaine treatment, and two weeks after ketamine treatment). Using this approach, we identified 65 genes that were significantly differentially expressed (likelihood ratio test; Benjamini–Hochberg-corrected *q* ≤ 0.1) (Fig. 5). Gene set enrichment analysis of this list identified significant enrichment of ontologies associated with endothelial development, regulation of angiogenesis, vascular development and tissue morphogenesis. Of note, many of the top scoring genes are components of the extracellular matrix (ECM) or have been implicated in its remodelling, including: *Fn1*(ref. ^37^), *Mmp16*(ref. ^38^), *Trpv4*(ref. ^39^), *Tinagl1*(ref. ^40^), *Nostrin*^41^, *Cxcr4*(ref. ^42^), *Adgre5*(ref. ^43^), *Robo4*(ref. ^44^) and *Sema3g*^45^. Additionally, the differentially expressed gene set includes the immediate early genes (IEGs) *Fos*, *Junb*, *Arc* and *Dusp*. When we did not control for the psychedelic-specific psychoactive response (saline versus all drug conditions, including cocaine), we identified 39 differentially expressed genes (Benjamini–Hochberg-corrected *q* ≤ 0.15) (Extended Data Fig. 10); however, enrichment analysis identified no significant ontologies associated with this gene set, and only 6 genes (*Hspa12b*, *Sema3g*, *Eng*, *Flt4*, *Cavin1* and *Ube4b*) overlapped with the differentially expressed genes in the open state versus closed state dataset shown in Fig. 5. These results provide evidence that the shared ability of psychedelics to reopen the social reward learning critical period converges at transcriptional regulation of the ECM. On the basis of these findings, our working model (Fig. 6) posits that psychedelics act at a diverse array of binding targets (such as SERT, 5-HT_2A_R, NMDA and KOR), to trigger a downstream signalling response that leads to activity-dependent (perhaps via IEG-mediated coincidence detection) degradation of the ECM, which in turn is the permissive event that enables metaplasticity. In this model, transcriptional upregulation of ECM components (for example, FN1) and downregulation of ECM proteolytic enzymes (for example, MMP-16), reflects the homeostatic response to these long-lasting cellular changes. Together, these results demonstrate novel biological effects (behavioural, temporal, electrophysiological and molecular) that—similar to therapeutic effects—are shared across psychedelics.

## Conclusions

These studies provide a novel conceptual framework for understanding the therapeutic effects of psychedelics, which have shown significant promise for treating a wide range of neuropsychiatric diseases, including depression, PTSD and addiction. Although other studies have shown that psychedelics can attenuate depression-like behaviours^35,46–48^ and may also have anxiolytic^49^, anti-inflammatory^50^ and antinociceptive^51^ properties, it is unclear how these properties directly relate to the durable and context dependent therapeutic effects of psychedelics^4,6–8^. Furthermore, although previous in vitro studies have suggested that psychedelic effects might be mediated by their ability to induce hyperplasticity^52^, this account does not distinguish psychedelics from addictive drugs (such as cocaine, amphetamine, opioids, nicotine and alcohol) whose capacity to induce robust, bidirectional, morphological and physiological hyperplasticity is thought to underlie their addictive properties^12^. Moreover, our ex vivo results (Fig. 4 and Extended Data Fig. 6) are consistent with in vivo studies, which demonstrate that dendritic spine formation following administration of psychedelics is both sparse and context dependent^47,53,54^, suggesting a metaplastic rather than a hyperplastic mechanism. Indeed, previous studies have also directly implicated metaplasticity in the mechanism of action of ketamine^55–57^. At the same time, since our results show that psychedelics do not directly modify addiction-like behaviours (Extended Data Fig. 4 and ref. ^11^), they provide a mechanistic clue that critical period reopening may be the neural substrate underlying the ability of psychedelics to induce psychological flexibility and cognitive reappraisal, properties that have been linked to their therapeutic efficacy in the treatment of addiction, anxiety and depression^58–60^.

Although the current studies have focused on the critical period for social reward learning, critical periods have also been described for a wide variety of other behaviours, including imprinting in snow geese, song learning in finches, language learning in humans, as well as brain circuit rearrangements following sensory or motor perturbations, such as ocular dominance plasticity and post-stroke motor learning^61–65^. Since the ability of psychedelics to reopen the social reward learning critical period is independent of the prosocial character of their acute subjective effects (Fig. 1), it is tempting to speculate that the altered state of consciousness shared by all psychedelics reflects the subjective experience of reopening critical periods. Consistent with this view, the time course of acute subjective effects of psychedelics parallels the duration of the open state induced across compounds (Figs. 2 and 3). Furthermore, since our results point to a shared molecular mechanism (metaplasticity and regulation of the ECM) (Figs. 4–6) that has also been implicated in the regulation of other critical periods^55–57,64,66^, these results suggest that psychedelics could serve as a ‘master key’ for unlocking a broad range of critical periods. Indeed, recent evidence suggests that repeated application of ketamine is able to reopen the critical period for ocular dominance plasticity by targeting the ECM^67,68^. This framework expands the scope of disorders (including autism, stroke, deafness and blindness) that might benefit from treatment with psychedelics; examining this possibility is an obvious priority for future studies.

## Methods

### Mice

Male wild-type mice were bred in house and weaned at 3 weeks or obtained from Jackson Laboratories (stock no. 000664). β-arr2-KO mice (stock no. 011130) were obtained from Jackson Laboratories, bred in house and weaned at 3 weeks of age. All mice were inbred to the C57BL/6J congenic ‘wild-type’ strain (as opposed to outbred ‘true wilds’, which were not used in this study). Congenic strains are generated by backcrossing for a minimum of 10 generations, a standard that is derived from the congenic interval, and the theoretical estimate that by the 10th generation, 99.99% of the congenic strain background will be from the recipient inbred^69^. Although the β-arr2-KO mouse (Jackson Laboratories stock no. 011130), was originally derived on the 129X1/SvJ background^70^, it was backcrossed to the C57BL/6J congenic strain at Jackson Laboratories (https://www.jax.org/strain/011130). All mice were maintained on a 12 h:12 h natural light:dark cycle, starting at 07:30 with food and water provided ad libitum. All behavioural experiments were conducted during the same circadian period (07:30–19:30) in a dedicated, sound- and odour-controlled behavioural testing room, which is separated from the vivarium, and no other experiments were conducted simultaneously in the same room. Sample size was estimated based on previous work and published literature. Experimenters were blind to the condition when subjective criteria were used as a component of data analysis, and control and test conditions were interleaved. Mice were randomly assigned to experimental and control groups. All procedures complied with the animal care standards set forth by the National Institutes of Health and were in accordance with protocols approved by the Johns Hopkins University Animal Care and Use Committee.

### sCPP assay

The protocol for sCPP was adapted from previously published work^11^. Mice were socially housed (3–5 males) in a cage containing corncob bedding (Anderson Cob, 0.25 inch cob, Animal Specialties and Provisions) until the pre-determined age for sCPP testing. Each mouse was used for only one behavioural time point. At the pre-determined age, mice were placed in an open field activity chamber (ENV-510, Med Associates) equipped with infrared beams and a software interface (Activity Monitor, Med Associates) to monitor the position of the mouse. The apparatus was partitioned into two equally sized zones using a clear Plexiglas wall, with a 5 cm diameter circular hole at the base; each zone contained one type of novel bedding (Alpha-Dri, Animal Specialties and Provisions or Kaytee Soft Granule, Petco). The amount of time spent freely exploring each zone was recorded during 30-min test sessions. For example, a score of 900 means that the mouse spent exactly 50% of its time on each of the two beddings, whereas a score of 1,800 means that it spent the full 30 min in the bedding that would be subsequently assigned as the social conditioning cue, and no time in the bedding that would be assigned as the isolation conditioning cue. After an initial pre-conditioning trial to establish baseline preference for the two sets of bedding cues, mice were assigned to receive social conditioning (with cage mates) for 24 h on one type of bedding, followed by 24 h of isolation conditioning (without cage mates) on the other bedding cue. To assure unbiased design, chamber assignments were counterbalanced for side and bedding cues. Immediately after the isolation conditioning, a 30-min post-conditioning trial was conducted to establish preference for the two conditioned cues. CPP is a learned association between a condition (for example, social) and a cue (bedding). It does not require scent from the other mice, as the bedding itself serves as the cue. Exclusion criteria for this behaviour are strictly defined as a pre-conditioning preference score of >1.5 or <0.5. Mice are never excluded based on the quality of their social interactions. Pre-conditioning versus post-conditioning social preference scores were considered significant if paired Student’s *t*-test *P* values were less than 0.05. Comparisons between experimental conditions were made using both normalized social preference scores (time spent in social zone post-treatment divided by pre-treatment) and subtracted social preference scores (time spent in social zone post minus pre); these were considered significant if unpaired Student’s *t*-test *P* values were <0.05. All experiments were performed during the mouse rest period (light cycle), since pilot experiments revealed that sCPP is most robust if assayed during this period. Prior to i.p. drug treatment experiments (MDMA, LSD, psilocybin, ketamine or ibogaine hydrochloride), mice were habituated to the injection procedure with daily saline i.p. injections in the home cage. Pharmacological delivery schedules were counterbalanced for type of drug. Unless otherwise stated (Fig. 2 and Extended Data Fig. 5), for pretreatment, experiments mice were tested 48 h after the injection to allow for complete clearance of the drug. For the experiment testing involvement of the 5-HT_2A_R, the 5-HT_2A_R antagonist ketanserin was administered i.p. 30 min prior to the injection of the drug tested.

### Electrophysiology

Subjects received an i.p. injection of either LSD (1 µg kg^−1^), ketamine (3 mg kg^−1^), psilocybin (0.3 mg kg^−1^), MDMA (10 mg kg^−1^), ibogaine (40 mg kg^−1^) or saline. Forty-eight hours after drug treatment, either parasagittal slices containing the NAc core (250 µm thick) or coronal slices containing the PL/IL region of the mPFC (250 µm thick) were prepared from C57BL/6 mice using standard procedures. In brief, after mice were anaesthetized with isoflurane and decapitated, brains were quickly removed and placed in ice-cold low-sodium, high-sucrose dissecting solution (228 mM sucrose, 26 mM NaHCO_3_, 11 mM glucose, 2.5 mM KCl, 1 mM NaH_2_PO_4_, 1 mM MgSO_4_, 0.5 mM CaCl_2_). Slices were collected with a Leica VT 1200s vibrating microtome. Slices were allowed to recover for a minimum of 60 min in a submerged holding chamber (∼25 °C) containing artificial cerebrospinal fluid (ACSF) consisting of 119 mM NaCl, 2.5 mM KCl, 2.5 mM CaCl_2_, 1.3 mM MgCl_2_, 1 mM NaH_2_PO_4_, 11 mM glucose and 26.2 mM NaHCO_3_. For hyperplasticity recordings (Extended Data Fig. 6), slices were removed from the holding chamber and placed into the recording chamber, where they were continuously perfused with oxygenated (95% O_2_, 5% CO_2_) ACSF at 2 ml min^−1^ at 25 °C. For metaplasticity recordings (Fig. 4), slices were removed from the holding chamber and incubated first for 10 min in oxygenated ACSF containing picrotoxin (50 µM, Sigma), followed by 10-min incubation in oxygenated ACSF containing both picrotoxin and oxytocin (1 µM, Tocris) before being placed into the recording chamber. Whole-cell voltage-clamp recordings from MSNs or layer V pyramidal cells were obtained under visual control using a 40× objective. The NAc core was identified by the presence of the anterior commissure, and the PL/IL region of the mPFC was identified by the presence of the forceps minor of the corpus callosum. Recordings were made with electrodes (2.5–4.0 MΩ) filled with 115 mM CsMeSO_4_, 20 mM CsCl, 10 mM HEPES, 0.6 mM EGTA, 2.5 mM MgCl, 10 mM sodium phosphocreatine, 4 mM sodium ATP, 0.3 mM sodium GTP and 1 mM QX-314. Miniature EPSCs were collected at a holding potential of −70 mV in the presence of tetrodotoxin (0.5 μM, Tocris Biosciences) and picrotoxin (50 μM, Sigma). Two minutes after break-in, 30-s blocks of events (total of 200 events per cell) were acquired and analysed using the Recording Artist plugin in Igor Pro software with threshold parameters set at 5 pA amplitude and <3 ms rise time. All events included in the final data analysis were verified visually. Data were analysed by multivariate analysis of variance (MANOVA) with three independent variables (drug, brain area and age) and two dependent variables (frequency and amplitude). Likelihood ratio test performed comparing the full model using treatment, age, and structure to a reduced model using age and structure. All calculations were performed in either GraphPad Prism 9 or the R programming language and are available as Supplementary Code 1 and in the repository at https://github.com/genesofeve/DolenPsychedelicOpenState.

### RNA extraction and sequencing

Male wild-type C57BL6/J mice were injected i.p. with LSD, ketamine, cocaine (20 mg kg^−1^) or saline solution either 2 weeks or 48 h before the mice were euthanized. At P98 to P112, mice were euthanized, brains were rapidly removed and ~1mm thick coronal slice (*n* = 3 mice per condition) containing the nucleus accumbens were sectioned using a mouse brain matrix. To microdissect the NAc, slices were placed in a petri dish containing ice-cold ACSF (125 mM NaCl, 2.5 mM KCl, 2 mM CaCl_2_, 1 mM MgCl_2_, 1.25 mM NaH_2_PO_4_, 10 mM glucose and 26 mM NaHCO_3_) supplemented with RNase inhibitor and oxygenated with carbogen gas (95% O_2_ and 5% CO_2_) to pH 7.3–7.4. The NAc was identified using the anterior commissure and other structural markers. Between each dissection, blades were replaced and all the instruments and the matrix were cleaned with a solution containing RNase inhibitor. Following dissection, tissue was immediately placed into 0.5 ml Trizol and subjected to a 15 s burst with a tissue homogenizer to lyse the cells. Samples were kept on ice prior to storage at −20 °C. Total RNA were extracted using the RNeasy Kit from Qiagen. The quality of purified RNA was assessed via both a nanodrop and 2100 Bioanalyzer from Agilent. Library preparation was performed using a TruSeq Stranded mRNA kit (Illumina) using the recommended protocol. Individual dual-indexed libraries were quality controlled, pooled, and sequenced on the NovaSeq 6000 platform on a single S1 flowcell to an average depth of 76,841,745 (±8,066,939.82) paired-end 100 bp reads per sample. Reads were pseudoaligned to the mouse GENCODE vM25 (ref. ^71^) reference transcriptome using kallisto (v0.46.2) with 100 bootstrapped samples and 6 threads. Defaults were used for all other parameters. Estimated transcript-level abundances were collapsed to gene-level expression estimates and analysed using the sleuth (v0.30.0) R/Bioconductor package. To identify genes with differential expression as a function of samples where the critical period is reopened we performed a likelihood ratio test comparing a full model which included batch, and critical period to a reduced model that only included batch. Time was not used as an explanatory variable in this model fitting. Using this test, we identified 65 genes as significantly differentially expressed at a 10% false discovery rate (Benjamini–Hochberg-corrected *q* ≤ 0.1). To identify genes with differential expression as a function of any drug treatment (including cocaine) versus saline we performed a likelihood ratio test comparing a full model that included batch, and ‘treated vs untreated’ to a reduced model that only included batch. Using this test, we identified 39 genes as significantly differentially expressed at a 15% false discovery rate (Benjamini–Hochberg-corrected *q* ≤ 0.15). Time was not used as an explanatory variable in this model fitting. Raw data will be made publicly available (Gene Expression Omnibus accession numbers: GSE230679 and GSM7231202–GSM7231228). Code to reproduce the RNA-seq analysis and associated figures is provided as Supplementary Code 2 and in the repository at https://github.com/genesofeve/DolenPsychedelicOpenState.

### Statistics

All statistical details can be found in the figure legends, including the type of statistical analysis used, *P* values, *n*, degrees of freedom, *t* values and *f* values. Sample sizes were not predetermined by statistical methods; instead they were estimated based on the previously published literature^11^. Data distributions were assumed to be normal. Homogeneity of variance was tested using Levene’s test for equality of variances. Comparisons between experimental manipulations were made using a two-tailed Students *t*-test (paired or unpaired, and with or without Welch’s correction as appropriate) and MANOVA for comparisons between multiple outcome measures, with *P* < 0.05 considered significant.

Linear, β-spline, loess smoothing and natural spline models evaluated on the previously published time course of normalized social preference scores^11^. Loess smoothing yielded a pseudoinverse at age 41.695 and a knot point of 35 was chosen for both β-spline and natural spline models. The natural spline outperformed the β-spline (adjusted *R*^2^ of 0.1053 versus 0.5554, respectively) with fewer parameters. Residuals were plotted against fitted values and age to check model assumptions. Leave one out cross validation was also used to assess model fit. Control data from all new experiments was used as test data via the predict R function. RSME and *R*^2^ values were comparable between the original model and the new data. Two-way *t*-tests to compare means of controls groups against matched or binned time periods was done to confirm fit to new data. The full model including coefficients for splines, experiment and condition was constructed and tested against reduced models with the final reduced model being reported. MANOVA analysis was carried out using multivariate linear models and the ANOVA function. All statistical comparisons were carried out in the R programming language and can be found in Supplementary Codes 3 and 4 as well as in the repository at https://github.com/genesofeve/DolenPsychedelicOpenState.

### Reporting summary

Further information on research design is available in the Nature Portfolio Reporting Summary linked to this article.

## Online content

Any methods, additional references, Nature Portfolio reporting summaries, source data, extended data, supplementary information, acknowledgements, peer review information; details of author contributions and competing interests; and statements of data and code availability are available at 10.1038/s41586-023-06204-3.

### Supplementary information

Reporting Summary

Supplementary Code Code to reproduce all the analyses in this Article. This version was frozen at time of resubmission. Subsequent versions, reformatted for increased accessibility, can be found at https://github.com/genesofeve/DolenPsychedelicOpenState. This project is licensed under the terms of the MIT license. Source Code 1 (MANOVA analysis relating to Fig. 4 and Extended Data Fig. 6), Source Code 2 (RNA sequencing analysis relating to Fig. 5 and Extended Data Fig. 10), Source Code 3 and Source Code 4 (regression analysis for Figs. 1 and 2 and Extended Data Figs. 2, 3, 5 and 7–9).

## Data Availability

Raw data are publicly available on the repository at https://github.com/genesofeve/DolenPsychedelicOpenState and at the Gene Expression Omnibus (accession numbers GSE230679 and GSM7231202 to GSM7231228).
