# Supplementary material for: Psychedelics reopen the social reward learning critical period
Source: Nature. 2023 Jun 14;618(7966):790–8. doi: 10.1038/s41586-023-06204-3 (PMC10284704; doi:10.1038/s41586-023-06204-3)
Supplement: Supplementary file 1 — Reporting Summary [file 41586_2023_6204_MOESM1_ESM.pdf]

nature portfolio

Corresponding author(s): Gul Dolen

Last updated by author(s): Apr 23, 2023

Reporting Summary

Nature Portfolio wishes to improve the reproducibility of the work that we publish. This form provides structure for consistency and transparency in reporting. For further information on Nature Portfolio policies, see our Editorial Policies and the Editorial Policy Checklist.

Statistics

For all statistical analyses, confirm that the following items are present in the figure legend, table legend, main text, or Methods section.

n/a	Confirmed	
☐	☒	The exact sample size (*n*) for each experimental group/condition, given as a discrete number and unit of measurement
☐	☒	A statement on whether measurements were taken from distinct samples or whether the same sample was measured repeatedly
☐	☒	The statistical test(s) used AND whether they are one- or two-sided<br>*Only common tests should be described solely by name; describe more complex techniques in the Methods section.*
☐	☒	A description of all covariates tested
☐	☒	A description of any assumptions or corrections, such as tests of normality and adjustment for multiple comparisons
☐	☒	A full description of the statistical parameters including central tendency (e.g. means) or other basic estimates (e.g. regression coefficient) AND variation (e.g. standard deviation) or associated estimates of uncertainty (e.g. confidence intervals)
☐	☒	For null hypothesis testing, the test statistic (e.g. *F*, *t*, *r*) with confidence intervals, effect sizes, degrees of freedom and *P* value noted<br>*Give P values as exact values whenever suitable.*
☒	☐	For Bayesian analysis, information on the choice of priors and Markov chain Monte Carlo settings
☐	☒	For hierarchical and complex designs, identification of the appropriate level for tests and full reporting of outcomes
☐	☒	Estimates of effect sizes (e.g. Cohen's *d*, Pearson's *r*), indicating how they were calculated

Our web collection on statistics for biologists contains articles on many of the points above.

Software and code

Policy information about availability of computer code

Data collection	For behavioral experiments, the position of the mouse was monitored using a commercially available software interface (Activity Monitor, Med Associates).
Data analysis	All statistical comparisons were carried out in either GraphPad Prism 9 or the R programing language.

For manuscripts utilizing custom algorithms or software that are central to the research but not yet described in published literature, software must be made available to editors and reviewers. We strongly encourage code deposition in a community repository (e.g. GitHub). See the Nature Portfolio guidelines for submitting code & software for further information.

Data

Policy information about availability of data

All manuscripts must include a data availability statement. This statement should provide the following information, where applicable:

- Accession codes, unique identifiers, or web links for publicly available datasets
- A description of any restrictions on data availability
- For clinical datasets or third party data, please ensure that the statement adheres to our policy

All data obtained in this study are available from the corresponding author upon reasonable request. RNA sequencing raw data will be made publicly available at GEO XXX. Code to reproduce the RNA-Seq analysis and associated figures is provided in a supplementary file. All data and analysis code will be available at the following repository: https://github.com/genesofeve/DolenPsychedelicOpenState.

Field-specific reporting

Please select the one below that is the best fit for your research. If you are not sure, read the appropriate sections before making your selection.

☒ Life sciences ☐ Behavioural & social sciences ☐ Ecological, evolutionary & environmental sciences

For a reference copy of the document with all sections, see nature.com/documents/nr-reporting-summary-flat.pdf

Life sciences study design

All studies must disclose on these points even when the disclosure is negative.

Sample size	Sample size was determined based on our previous findings and the published literature
Data exclusions	Mice were excluded if they exhibited a pre-conditioning preference score > 1.5 or < 0.5. These criteria were established prior to testing.
Replication	In order to maximize data robustness, care was taken to use automated analysis protocols and validate all assays for inter-rater reliability.
Randomization	To assure unbiased design for behavioral experiments, chamber assignments were counterbalanced for side and place cues.Group allocations were randomized during data collection.
Blinding	Experimenters were blind to the condition when subjective criteria were used as a component of data analysis, and control and test conditions were interleaved.

Reporting for specific materials, systems and methods

We require information from authors about some types of materials, experimental systems and methods used in many studies. Here, indicate whether each material, system or method listed is relevant to your study. If you are not sure if a list item applies to your research, read the appropriate section before selecting a response.

Materials & experimental systems

n/a	Involved in the study	
☒	☐	Antibodies
☒	☐	Eukaryotic cell lines
☒	☐	Palaeontology and archaeology
☐	☒	Animals and other organisms
☒	☐	Human research participants
☒	☐	Clinical data
☒	☐	Dual use research of concern

Methods

n/a	Involved in the study	
☒	☐	ChIP-seq
☒	☐	Flow cytometry
☒	☐	MRI-based neuroimaging

Animals and other organisms

Policy information about studies involving animals; ARRIVE guidelines recommended for reporting animal research

Laboratory animals	C57BL/6J mice were used for all experiments
Wild animals	Study did not involve wild animals
Field-collected samples	Study did not involve field-collected samples
Ethics oversight	All procedures complied with the animal care standards set forth by the National Institutes of Health and were in accordance with protocols approved by the Johns Hopkins University Animal Care and Use Committee.

Note that full information on the approval of the study protocol must also be provided in the manuscript.
